# Screening for Cardiovascular Risk in Asymptomatic Users of the Primary Health Care Network in Lebanon, 2012–2013

**DOI:** 10.5888/pcd11.140089

**Published:** 2014-07-17

**Authors:** Rouham Yamout, Salim M. Adib, Randa Hamadeh, Alia Freidi, Walid Ammar

**Affiliations:** Author Affiliations: Salim M. Adib, Faculty of Public Health, Lebanese University, Beirut, Lebanon; Randa Hamadeh, Ministry of Public Health, Beirut, Lebanon; Alia Freidi, Faculty of Health Sciences, American University of Beirut, Beirut, Lebanon; Walid Ammar, Faculty of Health Sciences, American University of Beirut, and Ministry of Public Health, Beirut, Lebanon.

## Abstract

**Introduction:**

In 2012, the Ministry of Public Health in Lebanon piloted a service of multifactorial cardiovascular screening in the publicly subsidized Primary Health Care (PHC) Network. We present an epidemiological analysis of data produced during this pilot to justify the inclusion of this service in the package of essential services offered through PHC and to present a preliminary cardiovascular risk profile in an asymptomatic population.

**Methods:**

A total of 4,205 participants (two-thirds of which were women) aged at least 40 years and reportedly free from diabetes, hypertension, dyslipidemia, and cardiovascular disease (CVD) were screened. The screening protocol used a questionnaire and direct measurements to assess 5 modifiable cardiovascular risk factors; total cardiovascular risk score was calculated according to a paper-based algorithm developed by the World Health Organization and the International Society of Hypertension.

**Results:**

Approximately 25% of the sample displayed metabolic impairments (11% for impaired blood glucose metabolism and 17% for impaired systolic blood pressure), and 6.6% were classified at total cardiovascular risk of 10% or more. Just over one-quarter of the sample was obese, almost half had a substantially elevated waist circumference, and 41% were smokers. Men were significantly more likely to screen positive for metabolic impairment than women, and women were more likely to be obese.

**Conclusion:**

The implementation of a multifactorial screening for CVD among asymptomatic subjects detected a substantial proportion of previously undiagnosed cases of high metabolic risk, people who could now be referred to optimal medical follow-up.

## Introduction

Lebanon has achieved its epidemiologic transition during the last 2 decades. Noncommunicable diseases became the leading cause of illness and death in Lebanon, accounting for 84% of all deaths in 2010 (1). Cardiovascular diseases (CVDs) alone account for almost half of all annual deaths in the country (2). Multiple factors contribute to CVD pathogenesis. Although some factors, such as age, male sex, and genetic predisposition to atherosclerotic disease, are nonmodifiable, others, such as tobacco use, poor weight control, alcohol abuse, hypertension, diabetes, and dyslipidemia, are amenable to modification and control (3).

Lebanon has a high prevalence of modifiable cardiovascular risk factors. For example, 54.6% of the population aged 25 to 65 are smokers (4). Self-reported diabetes has been estimated at 11% in 2008 (4), and hypertension has increased almost threefold in 1 decade (4,5).

Cardiovascular events are the clinical expression of atherosclerotic disease in major end organs, such as the heart (myocardial infarction) and brain (stroke) (6). The likelihood that someone will develop a fatal or nonfatal cardiovascular event depends on a combination of risk factors rather than on the presence of any single risk factor (7). Risk factors for CVD can be compiled in algorithms able to predict an individual’s total cardiovascular risk (TCVR) in a given period (8). The World Health Organization (WHO) in partnership with the International Society of Hypertension (ISH) developed charts adapted to the Eastern Mediterranean Region populations. The simplified version of the WHO/ISH charts does not include dyslipidemia and permits the stratification of 3 risk factors: systolic blood pressure (SBP), glucose metabolism, and smoking status, in addition to age and sex, to quantify a total 10-year cardiovascular risk score (9,10). The advantage of using these charts is the simplicity of their use and their low cost (11).

The Ministry of Public Health (MOPH) in Lebanon subsidizes secondary and tertiary health care for uninsured citizens in an effort to provide universal health coverage (12), although budgets allocated for preventive measures remain scarce and irregular (13). This approach of health governance contributed to a culture of overuse of advanced medical care (14) while the early detection of risk factors remains insufficient (15). This situation is illustrated by the considerable proportion of adults who have never had any blood glucose or blood pressure measurements (4), a situation that MOPH is trying to modify.

A pilot project was subsidized in the second half of 2012 to implement a standardized screening protocol for Primary Health Care Network (PHC) users without CVD for 5 cardiovascular risk factors and to estimate their TCVR using simplified WHO/ISH charts (16). The goal was to reach presumably healthy individuals, empower them with knowledge of their cardiovascular risk, and motivate them to seek medical attention and to choose measures to reduce that risk. We present an epidemiological analysis of data produced during this pilot phase to assess the usefulness of integrating the protocol in the routine services provided by PHC and to present a preliminary cardiovascular risk profile in an average healthy population.

## Methods

### Participants

The pilot project obtained data from a convenience sample of beneficiaries at 25 accredited PHC centers distributed across all Lebanese governorates (mohafazats), over 14 weeks during 2012 and 2013. Participants were recruited while present at the PHC centers (60% of participants) or through outreach visits to households in the PHC’s catchment area (40% of participants). Participants received clear information regarding the aims and objectives of the protocol and consented to be screened. They provided permission to use their answers and test results in anonymous analysis.

A total of 5,875 beneficiaries were screened. Records of only 4,205 were retained for analysis after excluding 1,279 (21.8%) cases previously diagnosed with diabetes, hypertension, dyslipidemia, or CVD. Records from 44 (0.7%) beneficiaries were excluded due to missing data on medical background, and 347 (5.9%) because of being younger than 40.

### Variables

#### Demographic and socioeconomic variables

Data for place of residence (urban or rural and mohafazat) were assimilated to the location of each health center. Data for employment status were self-reported and dichotomized into “involved in paid activity” full or part time and “not involved in paid activity” (including housewives and retired and unemployed persons). Data on educational level were also self-reported, and respondents were divided into 3 categories: illiterate, intermediate (those that had some schooling but not reaching the intermediate school exam), or secondary or higher. Beneficiaries of the pilot project were grouped into 4 age groups: 40 to 49, 50 to 59, 60 to 69, and 70 or older. Sex was designated as either male or female.

#### Cardiovascular risk factors

Risk factors measured in this project were those used for the WHO/ISH simplified risk score and others with a substantial contribution to CVD risk. Respondents were distributed into 5 categories of smoking: 1) occasional: at least 1 cigarette per day or at least 1 narghileh head per week or both, 2) current (daily) smokers, 3) quitters (within the last year), 4) ex-smokers (quit at least 1 year before the study), and 5) never smokers. This variable was further dichotomized into smokers, which included the first 3 categories, and nonsmokers, which included the last 2 categories. 

Random blood glucose (RBS) was measured using the OneTouch Select standardized finger-meter (LifeScan, Inc, Milpitas, California). Respondents were categorized as having “impaired RBS” (considered as participants with suspected diabetes) or “unimpaired RBS,” according to cutoff points that take into consideration the time of last meal, developed by the National Diabetes Program in Lebanon on the basis of International Diabetes Federation (IDF) guidelines and WHO recommendations (17,18). Beneficiaries with fasting blood glucose higher than 110 mg/dL, those with a blood glucose level higher than 125 mg/dL at least 2 hours after their last meal, and those with a blood glucose level higher than 135 mg/dL within 2 hours of their last meal were classified as having impaired blood glucose metabolism. 

SBP was measured on the right arm, over clothing, using standardized digital sphygmomanometers (Omron Healthcare, Hamburg, Germany). When a first measurement was abnormal, it was repeated later during the procedure and the lowest result was retained. Respondents were categorized as having impaired SBP if SBP was 135 mm Hg or more, according to the recommendations issued by MOPH. Participants’ body mass index (BMI) was grouped into 4 categories, according to WHO’s classification (19): less than 18.5 kg/m^2^ (underweight), 18.5 to 24.9 kg/m^2^ (normal weight), 25.0 to 29.9 kg/m^2^ (overweight), and 30.0 kg/m^2^ or higher (obese). Waist circumference (WC) was measured midway between the lower rib margin and the iliac crest with a regular tailor tape. According to the measurement, respondents were grouped into 3 categories: normal, elevated, and substantially elevated WC (SEWC). This categorization followed sex-specific cut-off points suggested by WHO and IDF: elevated WC indexed as a waistline measurement of 94 cm or more for men and 80 cm or more for women, and SEWC indexed as a waistline measurement of 102 cm or more for men and 88 cm or more for women (19).

### Total cardiovascular risk stratification

Impaired RBS, smoking status, and SBP scores were compiled by age and sex on the simplified WHO/ISH charts (10) to assign each screened participant a corresponding 10-year TCVR score category (Appendix). The compilation results were grouped in 5 predefined 10-year cardiovascular risk levels: less than 10% (lowest risk), 10% to less than 20%, 20% to less than 30%, 30% to less than 40%, and 40% or more (highest risk). The upper 3 risk categories were consolidated in the analysis because of small numbers. Sex, age, smoking status, impaired RBS, SBP, and BMI were also compiled on the Framingham online risk calculator using BMI instead of the lipid profile to project the 10-year mean risk of CVD (representing the 10-year incidence of CVD) (20,21).

### Procedures

After completing the structured questionnaire and undergoing measurements, beneficiaries of the pilot project were assigned a score on the WHO/ISH cardiovascular risk charts. Those with TCVR of 10% or more or with impaired SBP or impaired RBS or both were classified as “CV/metabolic risk.” These beneficiaries were invited to undergo further medical investigations including a lipid profile test and verification of abnormal screening results. The referral was usually made to the same health centers where normal though low fees had to be charged. Those who were metabolically unimpaired but were obese (BMI of 30.0 kg/m^2^ or more or SEWC) were advised to check their lipid profile, and smokers were given the option to undergo smoking cessation treatment.

### Data management and analysis

Trained health workers entered all data using a software developed for this purpose at MOPH-PHC. The data set was later read and analyzed using SPSS version 20 (IBM Corporation). Results were presented as frequencies and percentages. We used *z* tests and χ^2^ tests to assess associations in cross-tabulations. Significant associations were those with tests leading to a *P* value ≤. 05. 

## Results

### Sociodemographic characteristics

Of the 4,205 beneficiaries recruited in the pilot project, 59% were recruited from the PHC centers and the rest during household outreach from those centers (Table 1). Women represented almost two-thirds of the group; they had a mean age of 51 years, and almost 4 of 5 reported not being in the labor force. Men had a mean age of 52 years, and more than 80% were involved in paid activity. Approximately 13% of the group were illiterate, more than 25% reached at least secondary education, and the remainder had some schooling that did not exceed the 9th grade. Because of the predominance of women in the group and some significant differences between men and women found across several socioeconomic and demographic variables, all analyses were sex-stratified.

**Table 1:** Sociodemographic Characteristics of Participants^a^ (N = 4,205) in a Cardiovascular Screening Pilot Phase in Lebanon, 2012–2013

Variable	Men	Women	Total
	n (%)^b^		
**Sex**			
Male	—	—	1,422 (33.8)
Female	—	—	2,783 (66.2)
**Age, y**			
40–49	689 (48.5)*	1,556 (55.9)^†^	2,245 (53.4)
50–59	430 (30.2)*	763 (27.4)^†^	1,193 (28.4)
60–69	193 (13.6)*	303 (10.9)^†^	496 (11.8)
≥70	110 (7.7)*	161 (5.8)^†^	271 (6.4)
**Mean age, y (SD)**	52.2 (9.8)	50.8 (9.3)	51.3 (9.5)
**Work status (N = 4,174)**			
Involved in paid activity	1,180 (83.7)*	601 (21.7)^†^	1,781 (42.7)
Not involved in paid work	230 (16.3)*	2,163 (78.3)^†^	2,393 (57.3)
**Education (N = 4,069)**			
Illiterate	139 (10.0)*	393 (14.7)*	532 (13.1)
Up to intermediate	881 (63.5)*	1,616 (60.3)^†^	2,497 (61.4)
≥Secondary	367 (26.5)*	673 (25.1)^†^	1,040 (25.6)
**Place of examination**			
In Primary Health Care Network facility	809 (56.9)*	1,679 (60.3)^†^	2,488 (59.2)
Household outreach	613 (43.1)*	1,104 (39.7)^†^	1,717 (40.8)
**Residence, by centers’ location**			
Rural	864 (60.8)*	1,575 (56.6)^†^	2,439 (58.0)
Urban/suburban	558 (39.2)*	1,208 (43.4)^†^	1,766 (42.0)
**Mohafazat, by centers’ location**			
Beirut	134 (9.4)*	278 (10.0)*	412 (9.8)
Beirut suburb	115 (8.1)*	348 (12.5)^†^	463 (11.0)
Mont Lebanon	146 (10.3)*	225 (8.1)^†^	371 (8.8)
North Lebanon	295 (20.7)*	564 (20.3)*	859 (20.4)
South Lebanon	370 (26.0)*	724 (26.0)*	1,094 (26.0)
Nabatieh	167 (11.7)*	350 (12.6)*	517 (12.3)
Beqaa	195 (13.7)*	294 (10.6)^†^	489 (11.6)

a Values may not sum to 4,205 because of missing data.

b Data with different symbols (*, †) in columns of the same row denote significant difference in proportions between men and women; significance set at *P* ≤ .05 and calculated using *z* test.

### Prevalence of cardiovascular risk factors

The prevalence of smoking was 41% among respondents with a significant difference between men (47%) and women (38%; *P* < .001) (Table 2). Among smokers, 80.3% preferred cigarettes, 17.2% smoked narghileh only, and 1.6% smoked both cigarettes and narghileh (data not shown). More than 10% of beneficiaries (n = 457) screened positive for impaired RBS; more men (12.2%) than women (10.2%) had impaired RBS (*P* = .04). More than 15% of the participants had elevated SBP, significantly more among men (23.1%) than women (13.3%) (*P* < .001).

**Table 2:** Prevalence of Cardiovascular Risk Factors Among Participants in a Cardiovascular Screening Pilot Phase in Lebanon, 2012–2013

Variable	Men (n = 1,422)	Women (n = 2,783)	Total (N = 4,205)	*P* Value^a^
	n (%)			
**Smoking history**				
Never smoked	680 (47.8)	1,652 (59.4)	2,332 (55.5)	<.001
Ex-smokers	74 (5.2)	80 (2.9)	154 (3.7)	<.001
Smokers and those who quit within the last year	668 (47.0)	1,051 (37.8)	1,719 (40.9)	<.001
**Random blood glucose^b^**				
Unimpaired	1,248 (87.8)	2,500 (89.8)	3,748 (89.1)	.04
Impaired	174 (12.2)	283 (10.2)	457 (10.9)	
**Systolic blood pressure^c^**				
Unimpaired	1,094 (76.9)	2,412 (86.7)	3,506 (83.4)	<.001
Impaired	328 (23.1)	371 (13.3)	699 (16.6)	
**Body mass index, kg/m^2^**				
<25.0	419 (29.5)	913 (32.8)	1,332 (31.7)	.03
25.0–29.9	623 (43.8)	1,092 (39.2)	1,715 (40.8)	.004
≥30	380 (26.7)	778 (28.0)	1,158 (27.5)	.40
**Waist circumference**				
Normal	563 (39.6)	487 (17.5)	1,050 (25.0)	<.001
Elevated	433 (30.5)	675 (24.3)	1,108 (26.3)	<.001
Substantially elevated	426 (30.0)	1,621 (58.2)	2,047 (48.7)	<.001

Abbreviation: WC, waist circumference.

a *P* values calculated using χ^2^ test for dichotomous variables and using *z* test for variables with more than 2 categories.

b Participants with fasting blood glucose higher than 110 mg/dL, those with a blood glucose level higher than 125 mg/dL at least 2 hours after their last meal, and those with blood glucose level higher than 135 mg/dL within 2 hours of their last meal were classified as having impaired blood glucose metabolism.

c Participants were categorized as having impaired systolic blood pressure if pressure was 135 mm Hg or more.

More than a quarter of respondents (27.5%) were obese, and almost half of the respondents had a SEWC. Significantly more women (58.2%) than men (30.0%) had a SEWC (*P* < .001).

### Total cardiovascular risk

Of all beneficiaries from the screening protocol, 277 (6.6%) scored 10% or above for the TCVR score (Table 3); 9.7% of men (n = 138) and 4.8% of women (n = 139) (*P* < .01). To illustrate the potential effect of early cardiovascular risk-lowering management on the incidence of cardiovascular events, the sample’s characteristics were plotted using the Framingham risk calculators with BMI (Figure), modeling 2 situations. Model 1 represents the scores obtained by plotting the actual screening characteristics of the sample, and model 2 represents the hypothetical scores of the same sample after simulating the results of risk-lowering therapy on the 3 risk factors: 1) controlled blood pressure (SBP =120 mm Hg for those who scored higher), 2) a normal BMI (= 24.9 kg/m^2^ for those who were overweight or obese), and 3) a nonsmoking status (for those who reported being smokers). The projected risk was inferior to the actual risk by 35% in men and 41% in women.

**Table 3:** Distribution of Beneficiaries (N = 4,205), by Cardiovascular Risk Scores, Paticipants in a Cardiovascular Screening Pilot Phase in Lebanon, 2012–2013^a^

Sex/Age, y	Low TCVR Score, WHO/ISH Score <10%^b^		Moderate TCVR Score, WHO/ISH Score 10% to <20%^b^		High TCVR Score, WHO/ISH Score ≥20%^b^	
	n (%)	WHO^c^	n (%)	WHO^c^	n (%)	WHO^c^
**Men (n = 1,422)**						
40–49	686 (99.6)	99.1	2 (0.3)	0.7	1 (0.1)	0.2
50–59	413 (96.0)	83.9	9 (2.1)	7.8	8 (1.9)	8.3
60–69	145 (75.1)	34.6	33 (17.1)	28.5	15 (7.8)	27.9
≥70	40 (36.4)	8.6	48 (43.6)	34.2	22 (20.0)	57.2
Total men	1,284 (90.3)	NA	92 (6.5)	NA	46 (3.2)	NA
**Women (n = 2,783)**						
40–49	1,552 (99.7)	99.2	3 (0.2)	0.5	1 (0.1)	0.2
50–59	746 (97.8)	81.7	12 (1.6)	9.2	5 (0.7)	9.1
60–69	239 (78.9)	24.3	54 (17.8)	32.0	10 (3.3)	43.7
≥70	107 (66.5)	2.8	31 (19.3)	29.5	23 (14.3)	67.7
Total women	2,644 (95.0)	NA	100 (3.6)	NA	39 (1.4)	NA
**Total (N = 4,205)**						
40–49	2,238 (99.7)	NA	5 (0.2)	NA	2 (0.1)	NA
50–59	1,159 (97.2)	NA	21 (1.8)	NA	13 (1.1)	NA
60–69	384 (77.4)	NA	87 (17.5)	NA	25 (5.0)	NA
≥70	147 (54.2)	NA	79 (29.2)	NA	45 (16.6)	NA
**Total both sexes**	3,928 (93.4)	NA	192 (4.6)	NA	85 (2.0)	NA

Abbreviations: TCVR, total cardiovascular risk; WHO/ISH, World Health Organization/International Society of Hypertension; NA, not applicable.

a Men were significantly more likely to score 10% and above for TCVR (9.3% of men vs 5.2% of women, *P* < .001).

b TCVR assessed using simplified WHO/ISH charts (9,10).

c Projection of the distribution of the EMR region B population by WHO/ISH charts (6).

**Figure:**
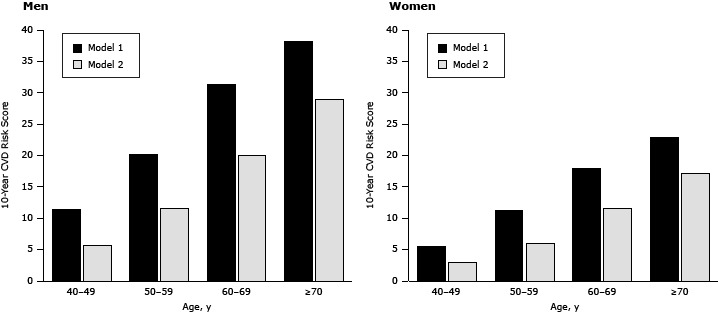
The means of 10-year general cardiovascular disease (CVD) risk scores calculated by using the Framingham equation (20, 21) with body mass index (model 1), and Model 1 plus modified risk factors (smoking, systolic blood pressure, and body mass index) (model 2), by age and sex. **Table:** 

Sex/Age, y	General Cardiovascular Risk Mean Score			
	Men		Women	
	Model 1	Model 2	Model 1	Model 2
40–49	11.4	5.8	5.5	3.0
50–59	20.2	11.7	11.2	6.0
60–69	31.4	20.0	18.0	11.6
≥70	38.3	29.0	22.9	17.2

### Cardiovascular risk score

According to screening results and TCVR scores by the WHO/ISH charts, 1,035 (25%) beneficiaries of the pilot project were referred to the diagnostic step. Of those, 277 (significantly more men than women, *P* < .001) had cardiovascular risk under the WHO/ISH definition with or without metabolic impairments; 758 had a low TCVR score but had impaired RBS, impaired SBP, or both, with an equal male predominance (*P* < .001).

Of 3,170 beneficiaries (75%) not eligible for referral, 1,536 were obese (SEWC or BMI ≥30.0 kg/m^2^), and 710 were not obese but were smokers (Table 4). In the group, 924 (22%) did not present any of the 3 modifiable risk factors considered. 

**Table 4:** Distribution of the Sample (N = 4,205), by Results of Screening and Eligibility for Referral to Follow-Up Steps, Participants in a Cardiovascular Screening Pilot Phase in Lebanon, 2012–2013

Cardiovascular Risk Groups	Men (n = 1,422), n (%)	Women (n = 2,783) , n (%)	Total (N = 4,205) , n (%)
**Referred to diagnostic step**	464 (32.6)	571 (20.5)	1,035 (24.6)
TCVR ≥10% and metabolic impairment	112 (7.9)	139 (5.0)	251 (6.0)
Only TCVR score ≥10%	26 (1.8)	0	26 (0.6)
Only metabolic impairment	326 (22.9)	432 (15.5)	758 (18.0)
**Not referred to diagnostic step**	958 (67.4)	2,212 (79.5)	3,170 (75.4)
Metabolically unimpaired obese^a^	306 (21.5)	1,230 (44.2)	1,536 (36.5)
Nonobese, metabolically unimpaired smokers	317 (22.3)	393 (14.1)	710 (16.9)
No risk factors detected	335 (23.6)	589 (21.2)	924 (22.0)

Abbreviation: TCVR, total cardiovascular risk.

a Indexed as body mass index ≥30 or substantially elevated waist circumference (waistline ≥94 cm in men and ≥88 cm in women) or both.

## Discussion

This pilot project used a simplified algorithm for the prediction of TCVR developed by WHO and ISH in 2009 and 2010 (9). WHO has encouraged individual countries to use this tool to identify patients who would benefit from medical attention to deal with their cardiovascular health (11). This injunction corresponded to a desire in Lebanon to implement a comprehensive CVD screening strategy focused on early detection of several cardiovascular risk factors in 1 procedure. The simplicity and the low-cost implementation of the charts were further incentives to use them as a mass screening tool. The running cost of the procedure did not exceed $0.50 per patient screened in the facility and $1 per patient screened in outreach. This inexpensive implementation was due to the voluntary contribution of nonphysician health workers employed at the centers of the PHC. The efficiency of this action in detecting a large number of potentially modifiable CVD risks in previously asymptomatic individuals was unequivocal, with 1 in 4 people detected for probable metabolic impairment and referred for further steps. The most important anticipated benefit from including such a protocol and implementing adequate follow-up for detected cases would be the decrease of CVD incidence, as demonstrated by the projection of the data using the Framingham algorithm.

PHC centers are more popular in rural areas than in urban areas. They attract beneficiaries from lower socioeconomic strata and are predominantly used by women. The sex distribution of the study population duplicates the sex distribution of PHC users in Lebanon (unpublished internal document of PHC, 2012). The greater use of centers by unemployed women may be partially caused by their availability during the morning hours when PHC centers are open. This observation calls for the modification of the hours that the PHC center is open to better accommodate working men (and women).

The proportion of people of lower socioeconomic status, as indicated by lower educational achievement (75%), was higher than the latest national figures for the same age groups (45%), reported in 2011 (22). The overrepresentation of less affluent people in the study’s population may have biased the findings, although it is not possible in the present phase of sociological transition in Lebanon to estimate whether the bias will be toward over- or under-estimating CVD risks for the entire population.

Men were more likely to be referred for metabolic impairment, which does not differ from global patterns of CVD (23). Women were more likely to have metabolically unimpaired obesity, reflecting the larger prevalence of obesity among women in the Middle East (24). The cutoff values for BMI and WC that were used may not be adapted for the Lebanese population, so the issue of defining optimal cutoff points and selecting the best obesity index to use should be addressed in forthcoming research.

The findings on RBS screening corroborate Lebanese national (25) and global (26) statistics, showing that diabetes is more likely to occur among men than women. However, the prevalence of hypertension among men and women is lower than that reported in national representative surveys conducted in 2005 (27) and 2008 (4) and the sex differential is larger. This particularity occurring over a short period is likely to be an artifact. 

The proportion of patients with moderate and high TCVR was less than that projected by WHO for the same age groups in the geographic subregion in which Lebanon is classified (6). This discrepancy is probably due to the inclusion in WHO projections of previously diagnosed metabolically impaired patients, a category that was excluded from our analysis. A better benchmark would be a comparison of figures from a more limited area in the region, emanating from interventions on apparently healthy people and using a similar procedure. Colleagues from Oman undertook a similar screening project but used different cutoff points for raised fasting blood glucose (≥100 mg/dL) and hypertension (≥130 mm Hg/≥85 mm Hg) (28). To make the comparison valid, we reclassified our patients according to fasting blood glucose and hypertension by using the same criteria used by the Omani colleagues and found comparable rates of positive screening results for both measurements (Table 5). However, the equivalence of findings resulting from the screening interventions cannot guarantee equality in overall prevalence of diabetes and hypertension in those 2 countries, nor does it eliminate the need for population-based prevalence surveys or continuous surveillance in Lebanon.

**Table 5:** Comparison of Findings of Pilot Project in Lebanon (2012) and the Pilot Screening in Oman (2008)^a^

Country/Sex	Hypertension (≥130/85 mm Hg)	Raised FBS (≥100 mg/dL)
	No. Screened (% With Condition)	
**Oman**		
Men	6,869 (36)	6,869 (44)
Women	11,043 (32)	11,043 (41)
Total	17,912 (33)	17,912 (42)
**Lebanon**		
Men	1,422 (43)	535 (44)
Women	2,783 (32)	1,006 (40)
Total	4,205 (36)	1,541(42)

Abbreviation: FBS, fasting blood glucose.

a Omani results from the Operational and Management Guidelines for the National Noncommunicable Disease Screening Program (28).

Several countries in the region engaged in successful implementation of CVD prevention on the basis of early detection of multiple cardiovascular risk factors, for example in Kuwait (29) and in Oman (28). However, Lebanon is so far the first country in the region to test and report on the implementation of TCVR score estimation with mass screenings conducted by nonphysician health workers inside PHC facilities and through outreach to beneficiaries’ homes. The added value of such an approach is dual. First, it maximizes the predictive value of the CVD screening by optimizing several easily and economically measurable cardiovascular risk factors (30). Second, using the risk stratification approach as an indicator rather than emphasizing results of each individual cardiovascular risk factor will help improve the awareness of health workers and beneficiaries on the joint adverse effects of clustering cardiovascular risk factors on cardiovascular health, and the importance of addressing all of them as a package (6–8).

## Appendix. World Health Organization/International Society of Hypertension (WHO/ISH) Risk Prediction Chart^a^ for EMR B Region, Displaying the 10-Year Risk Score of a Fatal or Nonfatal Cardiovascular Event (10)

**Table:**

Age, y	Men, %		Women, %		Systolic Blood Pressure, mm Hg
	Nonsmoker	Smoker	Nonsmoker	Smoker	
**EMR B People With Diabetes Mellitus**					

**70**	≥40	≥40	≥40	≥40	180
	≥40	≥40	≥40	≥40	160
	20 to <30	≥40	30 to <40	≥40	140
	10 to <20	20 to <30	10 to <20	20 to <30	120

**60**	≥40	≥40	≥40	≥40	180
	30 to <40	≥40	30 to <40	≥40	160
	10 to <20	20 to <30	10 to <20	30 to <40	140
	<10	10 to <20	10 to <20	10 to <20	120

**50**	30 to <40	≥40	≥40	≥40	180
	10 to <20	20 to <30	10 to <20	20 to <30	160
	<10	<10	<10	10 to <20	140
	<10	<10	<10	<10	120

**40**	10 to <20	30 to <40	10 to <20	20 to <30	180
	<10	<10	<10	<10	160
	<10	<10	<10	<10	140
	<10	<10	<10	<10	120

**EMR B People Without Diabetes Mellitus**					

**70**	≥40	≥40	≥40	≥40	180
	20 to <30	30 to <40	20 to <30	30 to <40	160
	10 to <20	20 to <30	10 to <20	20 to <30	140
	<10	10 to <20	<10	10 to <20	120

**60**	≥40	≥40	30 to <40	≥40	180
	10 to <20	20 to <30	10 to <20	20 to <30	160
	<10	10 to <20	<10	10 to <20	140
	<10	<10	<10	<10	120

**50**	20 to <30	≥40	20 to <30	≥40	180
	<10	10 to <20	<10	10 to <20	160
	<10	<10	<10	<10	140
	<10	<10	<10	<10	120

**40**	10 to <20	20 to <30	<10	10 to <20	180
	<10	<10	<10	<10	160
	<10	<10	<10	<10	140
	<10	<10	<10	<10	120

Abbreviation: EMR B, WHO Eastern Mediterranean Region, subregion B.

a This chart can be used only for countries of the EMR B: Bahrain, Iran (Islamic Republic of), Jordan, Kuwait, Lebanon, Libya, Oman, Saudi Arabia, Syrian Arab Republic, Tunisia, and United Arab Emirates.

## References

[1] World Health Organization. Cardiovascular diseases (CVDs). Fact sheet no. 317; 2013. http://who.int/mediacentre/factsheets/fs317/en/. Accessed February 14, 2013.

[2] Noncommunicable diseases country profiles 2011. Geneva (CH): World Health Organization; 2011.

[3] World Health Organization. Global status report on noncommunicable diseases, 2010. Geneva (CH): World Health Organization; 2012.

[4] Sibai A , Hwallah N . WHO STEPS chronic diseases risk factor surveillance. American University of Beirut; 2010. http://www.who.int/chp/steps/2008_STEPS_Le. Accessed February 14, 2013.

[5] National Household Health Expenditure and Utilization Survey (NHHEUS). The Lebanese Ministry of Public Health; 1999. http://www.pcm.gov.lb/Cultures/ar-LB/Menu. Accessed February 14, 2013.

[6] Prevention of cardiovascular disease: guidelines for assessment and management of cardiovascular risk. Geneva (CH): World Health Organization; 2007.

[7] Mendis S . Cardiovascular risk assessment and management in developing countries. Vasc Health Risk Manag 2005;1(1):15. 10.2147/vhrm.1.1.15.58933 17319094 PMC1993930

[8] Berger JS , Jordan CO , Lloyd-Jones D , Blumenthal RS . Screening for cardiovascular risk in asymptomatic patients. J Am Coll Cardiol 2010;55(12):1169–77. 10.1016/j.jacc.2009.09.066 20298922

[9] Package of essential noncommunicable (PEN) disease interventions for primary health care in low-resource settings. Geneva (CH): World Health Organization; 2010.

[10] Cardiovascular risk prediction charts for 14 WHO epidemiological sub-regions, page 21. World Health Organization, International Society of Hypertension; 2010. http://ish-world.com/downloads/activities/colour_charts_24_Aug_07.pdf. Accessed February 14, 2013.

[11] Cardiovascular risk prediction charts: strengths and limitations. World Health Organization, International Society of Hypertension. http://www.who.int/cardiovascular_diseases/publications/cvd_qa.pdf. Accessed February 14, 2013.

[12] Ammar W . Health system and reform in Lebanon. Geneva (CH): World Health Organization, Ministry of Public Health; 2010.

[13] Ammar W . Health beyond politics. Geneva (CH): World Health Organization, Ministry of Public Health; 2010.

[14] Sibai AM , Tohme RA , Saade GA , Ghanem G , Alam S , Lebanese Interventional Coronary Registry Working Group. The appropriateness of use of coronary angiography in Lebanon: implications for health policy. Health Policy Plan 2008;23(3):210–7. 10.1093/heapol/czn005 18356190

[15] Sfeir R . Strategy for national health care reform in Lebanon; 2007. http://www.fgm.usj.edu.lb/files/a62007.pdf. Accessed February 14, 2013.

[16] [Constituents/standard specifications and detailing services of the Primary Health care centres]. Ministry of Public Health, Lebanon; 2013. Arabic. http://www.moph.gov.lb/Prevention/PHC/Documents/HSServices.pdf. Accessed February 14, 2013.

[17] Diabetes atlas — updated guidelines for the definition, diagnosis, and classification of diabetes. Press conference, 19th World Diabetes Congress, Cape Town, South Africa. International Diabetes Federation; 2006.

[18] Definition and diagnosis of diabetes mellitus and intermediate hyperglycaemia: report of a WHO/IDF consultation. Geneva (CH): World Health Organization, International Diabetes Federation; 2006.

[19] Obesity: preventing and managing the global epidemic. Technical report series, 894. Geneva (CH): World Health Organization; 2000. 11234459

[20] D’Agostino RB , Vasan RS , Pencina MJ , Wolf PA , Cobain M , Massaro JM , Kannel WB . General cardiovascular risk profile for use in primary care: the Framingham Heart Study. Circulation 2008;117(6):743–53. 10.1161/CIRCULATIONAHA.107.699579 18212285

[21] The Framingham Health Study, interactive cardiovascular risk calculator. http://www.framinghamheartstudy.org/risk-functions/cardiovascular-disease/10-year-risk.php#. Accessed February 14, 2013.

[22] Yaacoub N , Badre L . Education in Lebanon, statistics in focus. Issue no. 3, Lebanon, tables. Central Administration of Statistics; 2013. http://www.cas.gov.lb/index.php/en/training-stat-en/83-english/images/Mics3/Tables/CAS_MICS3_9_Education.xls. Accessed February 14, 2013.

[23] Regitz-Zagrosek V , Lehmkuhl E , Weickert MO . Gender differences in the metabolic syndrome and their role for cardiovascular disease. Clin Res Cardiol 2006;95(3):136–47. 10.1007/s00392-006-0351-5 16598526

[24] Martorell R , Khan LK , Hughes ML , Grummer-Strawn LM . Obesity in women from developing countries. Eur J Clin Nutr 2000;54(3):247–52. 10.1038/sj.ejcn.1600931 10713748

[25] Sibai A , Obeid O , Batal M , Adra N , El Khoury D , Hwalla N . Prevalence of metabolic syndrome in Lebanese adult population: findings from the first epidemiological study. CVD Prev Contr 2006;3(2):83–90. 10.1016/j.precon.2007.06.002

[26] Diabetes atlas. 5th edition. Brussels (BE): International Diabetes Federation; 2011.

[27] Tohme RA , Jurjus AR , Estephan A . The prevalence of hypertension and its association with other cardiovascular disease risk factors in a representative sample of the Lebanese population. J Hum Hypertens 2005;19(11):861–8. 10.1038/sj.jhh.1001909 16034449

[28] Operational and management guidelines for the national noncommunicable disease screening program. Ministry of Health, Sultanate of Oman; 2010. http://www.moh.gov.om/en/reports/GuidelinesManual_for_the_national_NCD-screening_program.pdf. Accessed February 14, 2013.

[29] Alowaish R, Al Asi T, Adly L. Risk factors for cardiovascular diseases: Kuwait Heart Foundation’s mobile screening. J Saudi Heart Assoc 2012;24(4):295–6. 10.1038/sj.jhh.1001909 16034449

[30] Smith SC Jr , Greenland P , Grundy SM . AHA conference proceedings: prevention conference V: beyond secondary prevention: identifying the high-risk patient for primary prevention: executive summary: American Heart Association. Circulation 2000;(101):111–6. 10.1038/sj.jhh.1001909 10618313

